# Tryptophan Metabolism in Alzheimer’s Disease with the Involvement of Microglia and Astrocyte Crosstalk and Gut-Brain Axis

**DOI:** 10.14336/AD.2024.0134

**Published:** 2024-10-01

**Authors:** Lushuang Xie, Qiaofeng Wu, Kelin Li, Mohammed A. S. Khan, Andrew Zhang, Bharati Sinha, Sihui Li, Sulie L. Chang, David L. Brody, Mark W. Grinstaff, Shuanhu Zhou, Gil Alterovitz, Pinghua Liu, Xin Wang

**Affiliations:** ^1^Department of Neurosurgery, Brigham and Women’s Hospital, Harvard Medical School, Boston, MA 02115, USA.; ^2^Acupuncture and Moxibustion College, Chengdu University of Traditional Chinese Medicine, Chengdu, Sichuan, 610075, China.; ^3^Department of Chemistry, Boston University, Boston, MA 02215, USA.; ^4^Biomedical Cybernetics Laboratory, Department of Medicine, Brigham and Women’s Hospital, Harvard Medical School, Boston, MA 02115, USA.; ^5^Department of Biological Sciences, Institute of NeuroImmune Pharmacology, Seton Hall University, South Orange, NJ 07079, USA.; ^6^Department of Neurology, Uniformed Services University of the Health Sciences, Bethesda, MD 20814, USA.; ^7^Harvard Medical School, Harvard Stem Cell Institute, Boston, MA 02115, USA.

**Keywords:** Age-dependent Alzheimer’s disease;, tryptophan metabolism, microglia and astrocyte crosstalk, microbiota-gut-brain axis, bioinformatics analysis

## Abstract

Alzheimer’s disease (AD) is an age-dependent neurodegenerative disease characterized by extracellular Amyloid Aβ peptide (Aβ) deposition and intracellular Tau protein aggregation. Glia, especially microglia and astrocytes are core participants during the progression of AD and these cells are the mediators of Aβ clearance and degradation. The microbiota-gut-brain axis (MGBA) is a complex interactive network between the gut and brain involved in neurodegeneration. MGBA affects the function of glia in the central nervous system (CNS), and microbial metabolites regulate the communication between astrocytes and microglia; however, whether such communication is part of AD pathophysiology remains unknown. One of the potential links in bilateral gut-brain communication is tryptophan (Trp) metabolism. The microbiota-originated Trp and its metabolites enter the CNS to control microglial activation, and the activated microglia subsequently affect astrocyte functions. The present review highlights the role of MGBA in AD pathology, especially the roles of Trp *per se* and its metabolism as a part of the gut microbiota and brain communications. We (i) discuss the roles of Trp derivatives in microglia-astrocyte crosstalk from a bioinformatics perspective, (ii) describe the role of glia polarization in the microglia-astrocyte crosstalk and AD pathology, and (iii) summarize the potential of Trp metabolism as a therapeutic target. Finally, we review the role of Trp in AD from the perspective of the gut-brain axis and microglia, as well as astrocyte crosstalk, to inspire the discovery of novel AD therapeutics.

## 1. Introduction

Alzheimer’s disease (AD) is an age-dependent progressive neurodegenerative disease [1]. About 1 in 9 people age 65 and older have AD. The percentage of people with Alzheimer's dementia increases with age: 5.0% of people age 65 to 74, 13.1% of people age 75 to 84, and 33.2% of people age 85 and older have Alzheimer's dementia. AD is closely associated with the aging process. There is no cure for AD, which may partially reflect the lack of a clear AD pathogenesis. AD is characterized by extracellular Amyloid-β (Aβ) deposition and intracellular Tau-containing neurofibrillary tangles accumulation. Indeed, reports confirm that Aβ and Tau activities in human brain homogenates from Alzheimer’s patients [2, 3] and microglia and astrocytes are moderators of Aβ clearance and degradation that play critical roles in AD pathology. Interestingly, astrocytic degeneration is present in a subset of AD human brain samples [4]. Aging itself is the primary risk factor for the development of AD. Other critical factors affecting AD pathogenesis include genetics, environment, lifestyle habits, sleep, apolipoprotein E’s effect, viral and bacterial infection, and microbiota [5]. Microbial metabolites regulate the communication between astrocytes and microglia. Although such communication as part of AD pathophysiology is not well known. Aging affects the composition of the gut microbiota and microglia activity in neurodegenerative disorders like AD. Gut microbiota modulation and the communication between astrocytes and microglia represent a promising therapeutic strategy to counteract the progression of AD. Furthermore, recent advances in the field of aging biology, molecular biology, chemistry, and bioinformatics provide significant molecular insights into AD pathogenesis and promising therapeutics.

One of the potential links in bilateral gut-brain communication is tryptophan (Trp) metabolism. Trp enters the body via digestion of proteins in the gut. Subsequently, the small intestine absorbs this gut-derived Trp and its metabolites, such as nicotinamide adenosine dinucleotide (NAD), and then releases it into the peripheral circulation where it is transported through the large neutral amino acid transporter system (LAT1), crossing the blood brain barrier (BBB), and entering the central nervous system (CNS). After reaching the CNS, Trp acts on the aryl hydrocarbon receptor (AHR) to control the activation of astrocyte and microglia as well as to stimulate production of quinolinic acid (QUIN) and kynurenic acid (KYNA), respectively. QUIN is a direct activator of N-methyl-D-aspartate (NMDA) receptors that modulate the generation of ROS, lipid peroxidation, and release/uptake of glutamate. In AD pathology, indoleamine 2,3-dioxygenase (IDO) activity and production of QUIN increases in response to Aβ1-42 peptide in human macrophages and microglia. Also, in human AD brain specimen, the senile plaques show greater immunoreactivity to IDO1 and QUIN along with the other substances from reactive microglia and astrocytes. KYNA antagonizes the NMDA receptor as well as inhibits alpha 7 acetylcholine receptors, which regulate pre-synaptic glutamate release. The levels of KYNA decrease in the serum, red blood cells, and lumbar cerebrospinal fluid in AD patients [6]. QUIN and KYNA are responsible for the crosstalk between microglia and astrocytes, producing neurotoxic and neuroprotective effects, respectively. The present review highlights the role of microbiota-gut-brain axis (MGBA) in AD pathology, especially the roles of Trp *per se* and Trp metabolism as part of the gut microbiota and brain communications. We discuss the role of: (i) Trp and its derivatives in microglia-astrocyte crosstalk from a bioinformatics perspective; (ii) glia polarization in the microglia-astrocyte crosstalk and the involvement and interactions of inflammatory factors in AD pathology; and (iii) Trp in the glia crosstalk of gut-brain axis and summarizes Trp metabolism as a potential therapeutic target.

### Tryptophan derivatives in AD

#### Role of indoleamine 2,3-dioxygenase (IDO)

**Figure 1.**
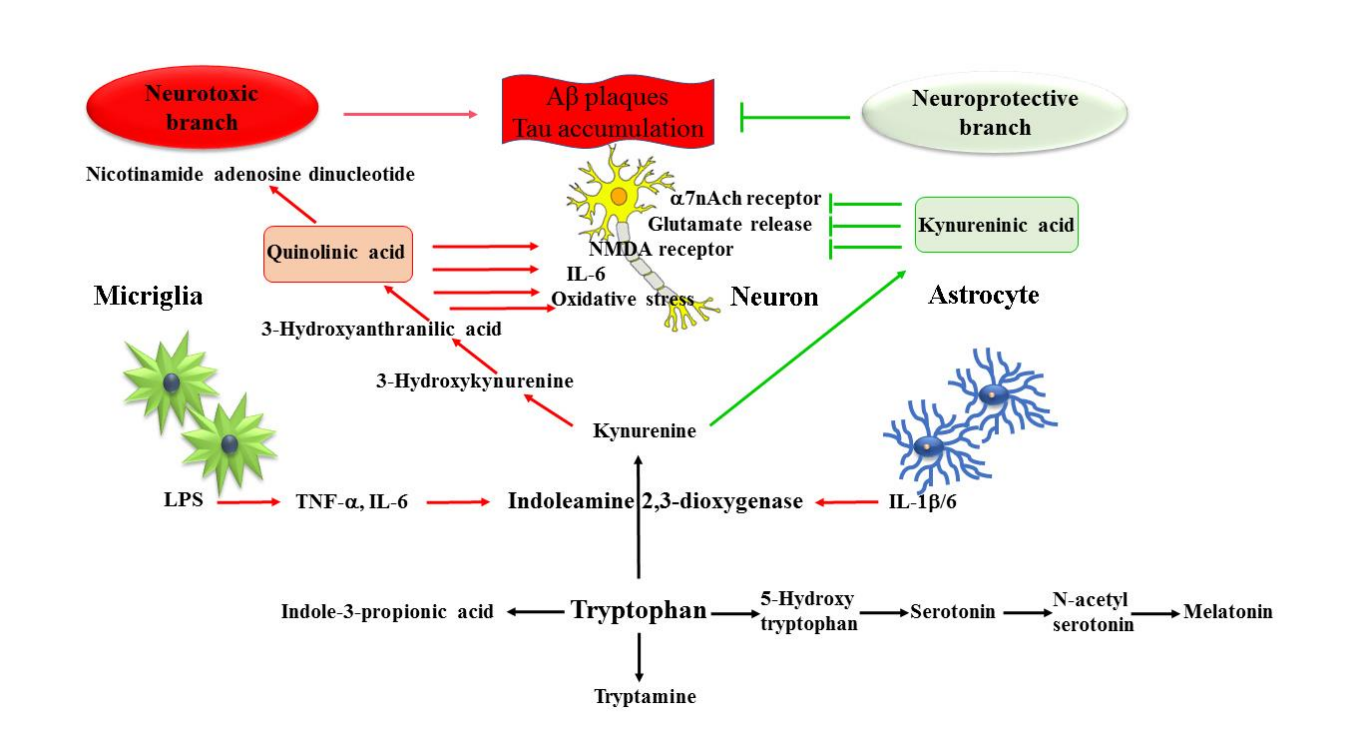
The metabolic routes of derivatives of tryptophan. The KYNA and QUIN pathways of major tryptophan (Trp) metabolism are depicted, which represent the neuroprotective branch and neurotoxic branch, respectively. The tryptophan-5-hydroxytryptophan (5-HTP), serotonin (5-HT), *N*-acetylserotonin (NAS), and melatonin pathways are shown as the minor pathways of Trp metabolism.

**Figure 2.**
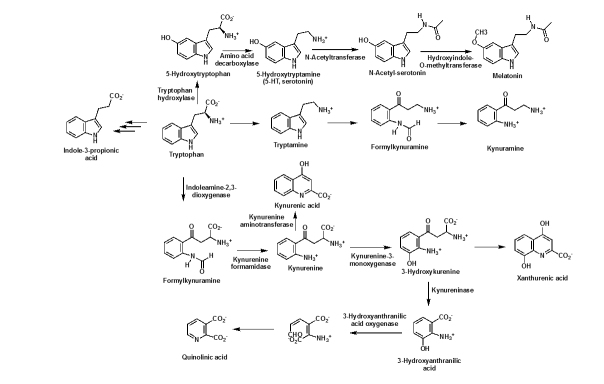
Scheme of the four Trp metabolic pathways. The kynurenine pathway to produce KYNA and QUIN; the hydroxylation pathway to produce 5-HTP-5-HT-NAS-melatonin; the decarboxylation pathway to produce tryptamine; and the transamination pathways to produce Indole-3-propionic acid.

Trp as well as its metabolism play vital roles in AD pathology. Trp metabolism is associated with aging and produces metabolites that control inflammation. The essential amino acid Trp is the precursor of serotonin (5-hydroxytryptamine (5-HT), which is widely distributed in both the peripheral nervous system (PNS) and the CNS [7]. There are four Trp metabolic pathways: (i) kynurenine (KYN), (ii) hydroxylation, (iii) decarboxylation, and (iv) transamination pathways. The major Trp metabolic pathway is the kynurenine pathway (KP), which produces various important Trp derivatives. The first step in the KP is the IDO-catalyzed Trp oxidation to formylkynurenine, which is then hydrolyzed to KYN [6, 8] (Fig. 1, 2). As the first and rate-limiting step in the KP of Trp metabolism, IDO is one of the potential players in AD pathogenesis. In 3xTg AD mice, IDO1 activity increases, which originates from the neurons and astrocytes [9]. Increased IDO activity leads to neuronal loss, and Tau protein, and Aβ accumulation via inflammatory pathways [7]. In a vicious cycle, IDO-mediated activation of inflammatory molecules give rise to pathological debris, which in turn increases the production of IDO [10]. Furthermore, the IDO inhibitor coptisine reduces serum IDO concentrations and leads to a decrease in microglia and astrocyte activation. As a result, coptisine treatment prevents neuron loss, reduces amyloid plaque formation, and ameliorates impaired cognition in the AβPP/PS1 transgenic AD mouse model [11]. Interestingly, both neurons and glia (astrocyte and microglia) produce IDO in the CNS [12]. Lipopolysaccharides (LPS) increase the production of IDO in microglia, leading to elevated levels of KYN along with an enhanced synthesis and secretion of the proinflammatory factors, tumor necrosis factor-α (TNF-α), and interleukin-6 (IL-6) [13] (Fig. 1). IDO causes loss of neurons, activation of glia, accumulation of debris, and an increase - in the proinflammatory factors and Trp depletion. These anomalies together lead to the enhanced progression of AD. Besides IDO, TDO (Tryptophan-2, 3-dioxygenase) is another heme-containing enzyme that catalyzes the production of KYN. TDO expression level is significantly raised in AD and leads to further enhancement of neuroinflammation and neuro-pathological abnormalities [14].

#### Roles of kynurenine (KYN) and kynurenic acid (KYNA)

Because KYN is one of the key intermediates after IDO catalyzed Trp oxidation in the KP pathway, the ratio of KYN/Trp is often used to evaluate IDO activation. AD patients possess elevated ratios of KYN/Trp [11, 15]. After KYN production, KYN metabolism diverges into two branches: (i) the neuroprotective branch and (ii) the neurotoxic branch (Fig. 1). By means of kynurenine aminotransferase II, KYN directly transforms into KYNA [16] (Fig. 1 and 2). Astrocyte primarily produce KYNA, which act as a neuroprotective agent in AD [17]. KYNA is an agonist of the AHR as well as acts as an endogenous antagonist of the NMDA receptor to prevent glutamate excitotoxicity in AD [6, 18, 19]. Astrocytic KYNA inhibits the α7 nicotinic acetylcholine receptors in neurons affording reduced levels of glutamate release (Fig. 1). KYNA-mediated interactions between astrocyte and neuron play a significant role in prefrontal mediated cognitive processes [20]. Glutamate serves as the energy source for neurons, and the shuttle of lactate between astrocytes and neurons can regulate the astrocyte glutamate uptake [21]. Due to these reasons, KYNA is a key Trp metabolic intermediate that mediates the neuron-astrocyte interaction, which regulates the extracellular glutamate concentration and related glutamatergic excitation process (Table 1). Besides KYNA-mediated astrocyte-neuron interactions, cytokines also serve as signals to connect the astrocytes and neurons. For example, interleukin-1β (IL-1β) is the core proinflammatory cytokine in AD, and astrocytic IL-1β causes an increase in Aβ accumulation in the neurons [22]. Blocking IL-1β production reduces the immune response [23, 24].

#### Role of quinolinic acid (QUIN)

On the neurotoxic branch, kynurenine 3-hydroxylase converts KYN to 3-hydroxykynurenine. Next, kynureninase transforms 3-hydroxykynurenine to 3-hydroxyanthranilic acid. Finally, 3-hydroxyanthranilate oxidase cleaves the aromatic ring and the resulting intermediate then re-cyclizes to produce QUIN. QUIN is the precursor of nicotinamide adenosine dinucleotide (NAD) [6, 8] (Fig. 1 and 2). The increase of the KYN/Trp ratio results in the elevation of QUIN [11, 15, 25]. QUIN and 3-hydroxykynurenine exhibit neurotoxic properties, and QUIN is involved in AD neuropathogenesis [26]. Although macrophages are the major producer of QUIN, microglia also produce QUIN but at a lesser quantity. QUIN produced by activated macrophages causes neurotoxicity through several pathways including activating NMDA receptors, inhibiting glutamate re-uptake, or increasing oxidative stress [6]. Increased QUIN is also present in peripheral mononuclear cells in Alzheimer's dementia [27].

**Table 1:** The pathology and function of derivatives of tryptophan in CNS.

Derivatives of Tryptophan	Mechanism	Pathology/Function	References
Kynurenic acid	Neuroprotection. The antagonist for NMDA receptors and cholinergic α7 nicotine receptors. Agonist of AHR. Targeting GRP35.	Reducing glutamate and proinflammatory cytokines release. Inhibiting glial inflammatory response. Reducing excitatory neurotransmission. Inhibiting hippocampal plasticity.	[17, 20, 61-63]
Quinolinic acid	Neurotoxin. Activating NMDA receptors, Iron chelation, and ROS production.	Increasing oxidative stress and cytosolic Cu/Zn SOD disturbance. Increasing lipid peroxidation activity in synaptosome. Increasing peroxidized lipids in the striatum and hippocampus, lactate dehydrogenase (LDH) release, and neuronal nitric oxide synthase. Upregulating IL-6. Impairing Spatial learning and memory and depression.	[6, 15, 26, 28, 30, 32, 33, 64, 65]
Serotonin	Serotonin neuron activity, neurotransmitter, Triggering serotonin receptor in astrocytes and microglia. Activating the HPA axis. Neuroprotection.	Improving brain development. Regulating the basic physiological functions (mood, sleep, pain, aggression and sexual behavior, cognition, memory, and anxiety).	[34, 66]
N-acetyl Serotonin	Neuroprotectant. Antioxidant. Anti-apoptosis.	Improving cognition and depression. Protecting against Aβ induced neurotoxicity in vitro. Increasing neuroprotection. Inhibition of caspase-3 activation. Anti-autophagy dysfunction. Reducing oxidative stress.	[46-49, 52]
Melatonin	Antioxidant, Antiinflammation, Triggering neurons or glia MT1/MT2 receptors. Neuroprotection.	Supporting mitochondria. Regulating circadian amplitudes. Increasing neuroprotection. Decreasing excitotoxic agents.	[35, 38, 39, 67]
Tryptamine	Inhibitor of BACE1, neurotransmitter, and neuromodulator. Neurotoxicity.	Increasing axonal loss. Damaging mitochondria. Causing neurodegeneration.	[55, 56, 68]
Indole-3-propionic acid	Neuroprotection. Antioxidant.	Reducing Aβ accumulation Decreasing oxidative stress. Reducing the levels of proinflammatory cytokines and the activation of glial cells and astrocytes. Inhibiting NF-κB signaling.	[55, 57, 60]

Additionally, IDO and QUIN are detected in cortical microglia, astrocytes, and neurons. In the perimeter of senile plaques of the AD human hippocampus, high levels of IDO and QUIN are present in microglia and astrocytes [28]. Microglia produce QUIN. Glia and immune cells express IDO, which leads to QUIN production and increased inflammatory response in CNS [15]. Increased QUIN production positively correlates with the formation of Aβ and p-Tau protein [26, 28]. Aβ induces IDO expression and leads to a significantly increased level of QUIN in human macrophages and microglia, while in human astrocytes and macrophages, Aβ induces IL-1β expression [29, 30]. QUIN impairs spatial learning and memory [26] and downregulating QUIN improves the IDO-dependent depressive-like behaviors in the hippocampus of LPS-treated mice [31]. Astrocytes and neurons could uptake external QUIN to produce NAD [25]. In explaining QUIN’s neurotoxicity, Schiefer *et al*. report a substantial IL-6 upregulation in the rat striatum and the loss of striatal neurons following QUIN injection [32] (Table 1).

QUIN plays an important role in AD pathology. Firstly, it induces the production and accumulation of neuropathological substances [26, 28] due to the increase in oxidative stress and neuroinflammation that enhance the neuronal damage [15, 26]. It also disorders the metabolism of lactate in neurons that results in derailment of a variety of neurotransmitters [33]. These neuro-pathological alterations by QUIN induce loss of memory and further the progression of AD.

### Role of melatonin

The KP accounts for 95% of Trp metabolism, and the other 3 minor pathways are responsible for 5% of Trp degradation [8, 34]. However, many important Trp metabolites are from the 3 minor pathways. For example, tryptophan hydroxylase hydroxylates Trp to produce 5-hydroxytryptophan, followed by conversion to serotonin by 5-hydroxytryptophan decarboxylase (Fig. 1 and 2). Next, serotonin *N*-acetyltransferase acetylates serotonin to afford *N*-acetylserotonin (NAS), which is methylated by hydroxyindole *O*-methyltransferase to produce melatonin as the final product [8, 35] (Fig. 1 and 2). Many of these molecules contribute to AD pathology and other neurological diseases [36].

Abnormal protein aggregates are the common pathological hallmarks for neurodegenerative diseases, including AD (Aβ peptides), Parkinson's disease (PD) (α-synuclein), Huntington's disease (HD) (Huntingtin), and Amyotrophic Lateral Sclerosis (ALS) (superoxide dismutase 1 or TAR-DNA binding protein-43). Melatonin, an anti-aging agent, as reported by us and others, supports the normal CNS function in HD through decreasing mutant huntingtin-mediated toxicity [37], and in AD through decreasing Aβ plaques, Tau protein hyperphosphorylation, supporting mitochondrial function and reducing oxidative stress and the inflammatory responses [38-41]. Furthermore, the neuroprotective effects of melatonin, another melatonin receptor agonist, arise through the upregulating of melatonin receptor type 1A (MT1) in HD [37] and ALS [42]. Interestingly, Sulkava *et al*. report higher levels of Aβ and Tau protein accumulations in AD patients with the rs12506228A variant, and this variant gene is located close to the MT1 gene. MT1 expression levels affect rs12506228A expression, while MT1 silencing leads to an elevated Aβ pathological process in culture neurons. Thus, MT1 is a gene responsible for regulating the amyloidogenic processing of amyloid precursor protein (APP) [39]. Mouse macrophage gene expression data, as analyzed by Kadena *et al*., show that melatonin attenuates immune responses and preferentially downregulates interferon regulatory factors, signal transducers, and activators of transcription-related signaling [43]. Recently, single-cell analysis and genetic fate mapping data reveal that a spectrum of macrophage/microglia exists in variety of phenotypic states. While the differentially expressed genes (DEGs) in the different macrophage phenotypes have been compared with the ImmGen database. The results demonstrate that both microglia and astrocyte display a multifunctional role in neuronal activity modulation in homeostatic and pathophysiological conditions [44].

### Role of N-acetylserotonin (NAS)

NAS is a Trp methoxyindole derivative and may function as a melatonin receptor agonist. NAS is also an agonist of the tyrosine protein kinase B and exhibits antioxidant and neurotrophic effects [45, 46]. In cerebellar granule cell culture, NAS protects against Aβ induced neurotoxicity (Table 1) [47]. Additionally, NAS offers neuroprotection in experimental models of ischemic stroke [48, 49], retinal ischemia-reperfusion [50], hepatic ischemia-reperfusion injury, and hepatic cell apoptosis [45, 51]. NAS is being explored in developing treatments for age-associated cognitive decline and depression [52, 47] (Table 1).

DEG analysis and gene set enrichment assay (GSEA) of brain tissues with different levels of serotonin show that serotonin downregulates lipid metabolism-related genes [53]. Because lipids are the key components of neuronal structure and brain development [54], results from these DEG and GSEA analyses support the beneficial role of serotonin in neurons. Contrary to the neuroprotective effects of serotonin, NAS, and melatonin, some Trp metabolites, *e.g*., 5-hydroxyindoleacetic acid, 5-methoxytryptophol, and 5-hydroxytryptophol, cause neurotoxicity [35].

### Roles of tryptamine and indole-3-propionic acid (IPA)

Decarboxylation and transamination reactions of Trp afford tryptamine and indole-3-propionic acid (IPA), respectively. IPA also can be transformed into KYNA [55, 56]. Tryptamine is an inhibitor of β-secretase, known as the β-site APP cleaving enzyme 1 (BACE1). The BACE1 enzyme is essential for the generation of β-amyloid. BACE1 catalyzes the initial cleavage of the APP to generate Aβ, which is the starting point of Aβ plaque formation [56]. Gut bacteria produce IPA from Trp. More specifically, IPA production completely depends on the presence of gut microflora and could be established by colonization with the bacterium *Clostridium sporogenes* [57]. The introduction of IPA producing enteric bacteria into the gastrointestinal tract is sufficient to introduce IPA into the host bloodstream [57]. Following absorption from the intestine and distribution to the brain, IPA offers a neuroprotective effect [58]. As a potent neuroprotective antioxidant and an inhibitor for Aβ fibril formation, IPA is prime for evaluation as a potential therapeutic for AD [59]. In humans, IPA treatment reduces Aβ accumulation levels and lessens oxidative stress [55]. IPA treatment also inhibits NF-κB signaling, reduces the levels of proinflammatory cytokines TNFα, IL-1β, and IL-6 in response to endotoxin in macrophages [60], and decreases the activation of glia and astrocytes. Taken together, Trp metabolic pathways are promising AD therapeutic targets.

### Microglia and astrocyte crosstalk in AD

#### Sketch of microglia and astrocyte

Microglia and astrocyte are the major immune response cells in the CNS. Normally, they are responsible for improving tissue repair, maintaining homeostasis, and mitigating proinflammatory activity and tissue destruction [69]. Microglia, which serve as the primary immune cells of the CNS, regulate the innate immune functions of astrocytes, and determine the roles of reactive astrocytes ranging from neuroprotective to neurotoxic [70]. The Aβ-induced microglia increase astrocyte reactivity to express associated activated genes, such as C3, and Ggta1. Interestingly, NLY01, a glucagon-like peptide-1 receptors (GLP-1R) agonist, inhibits the microglia-induced activation of astrocyte. NLY01 can block Aβ-induced activation of microglia through GLP-1R and blocks the reactivity of astrocytes as well as reduces the accumulation of Aβ and preserves neurons in AD mouse models [71]. Thus, providing an example of functional interactions between microglia and astrocytes in relation to targeting these glial cells for possible AD therapy.

Not only microglia regulate the functions of astrocytes, but also astrocytes in turn regulate microglia functions and phenotypes. The IL-3 originating from the astrocyte triggers the IL-3R of microglia that results in increased pathological debris clearance by the microglia [72]. The reactive astrocytes also produce C3 via the NF-kB pathway induced by Aβ. The receptor, C3R, abundantly exists in the microglia and binding to C3R promotes microglia phagocytosis. Interestingly, both astrocytes and microglia secret C3, which affords a more anfractuous process [73].

Given the significant role of glia in the process of AD, astrocytes and microglia are potential therapeutic targets. For example, non-steroidal anti-inflammatory drugs (NSAIDs), such as indomethacin and flurbiprofen, reduce the pathological debris products in AD, in which the NSAIDs inhibit the enhancement of proinflammatory response via altering the activation of glia [74]. Additionally, NLY01 acts as a GLP-1R agonist to relieve the neurodegenerative disorders, including AD and PD. NLY01 blocks the debris-induced microglia activation and reduces the accumulation of reactive astrocyte [71].

### Neuroinflammatory crosstalk of glia in AD

#### Activation of glia

**Figure 3.**
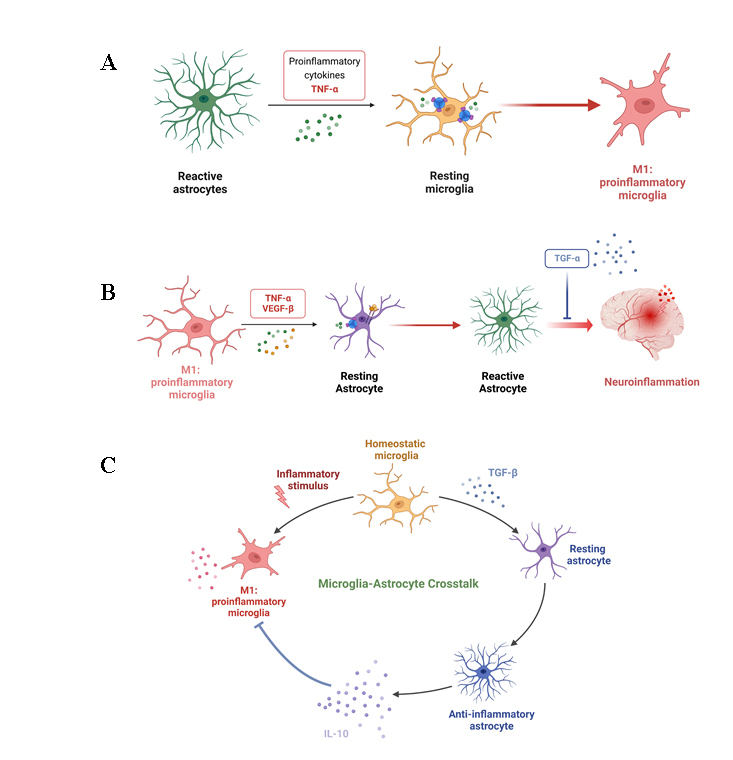
The interaction between microglia and astrocyte. (A) The reactive astrocyte promotes the activation of resting microglia via TNF-α, which switches them to proinflammatory microglia. (B) M1-type microglia induce the activation of resting astrocytes to convert them into reactive astrocyte, leading to neuroinflammation. In contrast, secretion of TGF-β inhibits neuroinflammatory response in the brain. The proinflammatory microglia induce neuroinflammation of astrocyte. (C) The circularity of interaction and crosstalk between microglia and astrocyte. This figure is created with BioRender.com.

Both microglia and astrocytes are major contributors of homeostasis in the CNS. The activation of microglia and astrocytes is the fundamental pathology in AD. However, dysregulation of normal function leads to cognitive deficits and neurodegeneration in pathogenesis of AD. Microglia can switch from the quiescent state to the disease-associated phenotype based on the analyses by both single-cell RNA sequencing (scRNA-Seq), as well as single-nucleus RNA sequencing, revealing subpopulations with distinct molecular and functional characteristics. Just like macrophage, the phenotypes of microglia also appear in different forms and are more complicated. For example, the disease-associated microglia (DAM) deliver a signal to recruit macrophages in the brain in neurological diseases, such as AD. In this disease, the DAM alters the gene expression of both homeostatic markers as well crucial pathological genes of cytokines and phagocytosis factors [75]. For instance, the gene expression of proliferation marker Ki67 changes in the 5xFAD model. In another example, TREM2 is a very important factor for microglial proliferation [76]. Furthermore, the microglia in AD can be separated into 4 types, DAM, IFN-I, MHC and proliferating types [77, 78]. Besides AD, microglia are also altered in other diseases or injuries of the CNS, such as Multiple sclerosis and traumatic brain injury. Additionally, a variety of microglial phenotypes exists in different brain regions as determined by a differential expression of genes. For example, CD68 and HLA-DR are highly expressed in white matter, while MHC-II is abundantly expressed in grey matter [79, 80]. Further, cytokines (*e.g.*, IL-1, -12, -17, -18 and -23), chemokines (*e.g.*, CCL-2, -12 and CXCL10) and other inflammatory molecules such as nitric oxide, reactive oxygen species, prostaglandin E2 and matrix metallopeptidase-9 and -10 are also significantly elevate in microglia during the neurotoxic phase. In contrast, alternatively activated subsets release anti-inflammatory cytokines (*e.g.*, IL-4, -10 and -13), chemokines (*e.g.*, CCL-17, -22, -24), and other neuroprotective molecules such as brain-derived neurotrophic factor, vascular endothelial growth factor (VEGF), insulin-like growth factor-1, nerve growth factor, and suppressor of cytokine signaling 3 when the microglia are in repairing and neuroprotective phase. There are two classes of astrocytes (i) protoplasmic astrocytes (which possess short projections, envelops synapses and present in the gray matter) and (ii) fibrous astrocytes (consisting of long projections having contact with Nodes of Ranvier located in the white matter). Like microglia, naïve/resting astrocytes also undergo reactive astrogliosis during inflammation and change their phenotype into three different forms depending on the pathological and recovering stages. The first type of astrocytes can switch to reactive astrocytes and become neurotoxic in neuroinflammatory conditions, causing impairment of synapses with increased level of C3. The second type of astrocytes (A2 reactive) adopt a neuroprotective status by repairing synapses with increased expression of S100 after ischemia. The third type belongs to the scar-forming astrocytes, which exhibit the characteristics of hypertrophy, cell proliferation with an overexpression of glial fibrillary acidic protein (GFAP) [81, 82]. However, the polarization and function of microglia are not categorical. The different phenotypes of microglia express a spectrum of activation patterns, not only absolute subtypes. Furthermore, the phenotypes of microglia are not stable, one phenotype can transform into another phenotype under the alteration of the surrounding microenvironment [81]. The activation and phenotypic transformation of astrocyte is microglia dependent. Released TNFα, IL-1β, and Complement component C1q (C1q) activate astrocytes through the downregulation of CXCR7 receptor and the PI3K-AKT pathway. In contrast, upregulating CXCR7 receptor by its agonist AMD3100 switches microglia and astrocytes into neuroprotective subtypes [82]. The gene transcriptome analyses of astrocytes show that 57 genes (*e.g.*, C, guanine nucleotide binding protein 2; Guanylate Binding Protein 2, histocompatibility 2, D region locus 1; H2-D1 and Serping-1) are associated with neurotoxic function and 110 genes (*e.g.*, S100 calcium-binding protein 10; pentraxin 3; sphingosine-1-phosphate receptor-3; and Tweak) are related to neuroprotective function of astrocytes [83]. In inflammatory conditions, the reactive astrocytes promote the activation of resting or quiescent microglia through the release of proinflammatory cytokines such as TNF-α changing their phenotype to proinflammatory microglia (Fig. 3A). In succession, these proinflammatory microglia activate resting astrocytes into reactive astrocytes that is modulated by the release of TNF-α and VEGF, leading to neuroinflammation. However, the production of anti-inflammatory cytokine TGF-α in the tissue microenvironment attenuates reactive astrocytes-mediated neuroinflammation (Fig. 3B). Normally, microglia maintain homeostatic environment in the CNS. The change in homeostatic status of the microglia solely depends on the stimuli, which can dictate microglia to either activate themselves to become proinflammatory type or switch to anti-inflammatory microglia and release TGF-β. The resting astrocytes, in response to the release of TGF-β change their state to anti-inflammatory astrocytes. In turn, these astrocytes produce IL-10 to reverse the proinflammatory state of the microglia. This crosstalk between microglia and astrocytes on the one hand initiates neuroinflammation and on the other hand resolves the neuroinflammatory response in the brain (Fig. 3C). Furthermore, the subtypes of glia depend on the glia situation in different physiology and pathology. For example, microglia express different features under different physiological or stimulated station, such as “satellite” microglia, Keratan sulfate proteoglycan (KSPG) microglia [84]. The subtypes of astrocytes express similar complexity because they play various functions in CNS). Astrocytes also vary in different regions of CNS. Results from a scRNA-Seq of large-scale integration analysis reveal that the different astroglia clusters distribute in different regions of the brain, such as cortex, hippocampus and midbrain. And the phenotypes of astrocytes also alter in different diseases, such as AD and PD [85]. The activation of AHR inhibits TNF-α expression in astrocytes, which indicates that AHR might be involved in astrocyte-mediated microglia polarization [86]. Acute hypoxia induces resting microglia polarization towards the proinflammatory phenotype through the activation of the NF-κB pathway, in which proinflammatory factors, *e.g*., TNF-α, IL-6, and CCL2, are increased, and antiinflammatory substances, *e.g*., IL-4 and IL-10, are decreased [87] (Fig. 2). The interaction between Trp and AHR increases TGF-α levels and decreases VEGF-β levels, via regulating the microglia NF-κB pathway, which is a primary inflammatory pathway [88].

The NF-κB pathway activation also upregulates TGF-α, which in turn promotes the glutamate transporter-1 production in astrocytes [89]. The reactivity of astrocyte is recognized as the one of basic pathological alterations of AD [90]. Glia cells, especially microglia and astrocyte, play a central role in orchestrating the neuroinflammation processes and neurodegeneration. Neuroinflammation serves as a bridge connecting microglia and astrocytes. Along these lines, dietary Trp metabolites, which are produced by microbiota in the gut, cross the blood brain barrier to activate microglia, which produces TGFα and VEGF-β by cross talking with astrocytes to change its transcriptional activity through AHR, subsequently modulating neuroinflammation [88]. The effects of Trp and its metabolites on phenotypic changes of microglia and astrocytes, and their crosstalk will clarify the roles of both glial cells in neurotoxicity and neuroprotection.

### Role of VEGF

In astrocytes, VEGF-β stimulation upregulates proinflammatory factors (Fig. 3), *e.g*., TNF-α, IL-6, and nitric oxide synthase 2 (NOS2), while TGF-α upregulates anti-inflammatory factors such as IL-10 [88]. Astrocytes express VEGF receptor Flk-1, which responds to the VEGF stimulation to alter the number and shape of astrocytic branching and the distance between astrocytes [91]. VEGF stimulation of astrocytes also promotes BBB infiltration, which allows the inflammatory factors to cross the BBB and enter the CNS [92]. VEGF-β stimulation also increases astrocytic NOS2 expression by blocking AHR [86, 88]. As one of the iNOS, NOS2 produces nitric oxide (NO) to regulate inflammation and development. Blocking astrocytic NOS2 inhibits cell development and differentiation [93]. LPS/IFN-γ treatment upregulates NOS2, like other proinflammatory cytokine genes (CCL2, IL-6), and LPS and IFN-γ are the typical stimuli leading to the microglia/macrophage proinflammatory phenotype [94]. iNOS is usually used as the marker for the proinflammatory phenotype, which is highly expressed in AD [94, 95]. Upon VEGF stimulation, the activated astrocytes produce proinflammatory cytokines IL-1β, TNF-α, and IL-6, which induce neurotoxicity in neurodegeneration [86, 88, 96]. The presence of Aβ elevates astrocytic inflammatory mediators (*e.g*., TNF-α, IL-1β, iNOS, and cyclooxygenase 2 (COX-2)) [97]. Then, these mediators enhance Aβ accumulation or Tau protein deposits which leads to increased neurotoxicity due to the reduction of glia phagocytosis [98-100]. For example, TNF-α enhances Aβ accumulation and the activation of the NF-κB pathways, which result in iNOS up-regulation and oxidative stress [101].

### Role of transforming growth factor-β (TGF-β)

Unlike VEGF, the TGF-β signal usually expresses anti-inflammatory action in astrocytes. Upregulation of the astrocytic TGF-β pathway inhibits immune cell accumulation, cytokine, and chemokine production [102]. As an anti-inflammatary factor to prevent AD, TGF-β expression level increases with age [103]. Astrocytic TGF-β inhibits the binding of Aβ to the synapses to maintain the synapse density. In AD, an increase in Aβ and loss of synapses are the primary hall markers [104]. Microglia originated-TGF-β stimulates astrocytes to produce IL-10, which is widely recognized as a protective factor to relieve inflammation [88, 105] (Fig. 3). Anti-inflammatory phenotype cells, such as microglia/macrophage, produce key anti-inflammatory cytokines (*e.g*., IL-10, IL-4, and IL-13) to relieve neuroinflammation [96, 105]. IL-4 and IL-13 protect against LPS-induced neuron injury by suppressing the production of proinflammatory cytokines (*e.g.*, IL-8, IL-6, and TNF-α) and by reducing NO release [96].

Although TGF-β has been traditionally recognized as an anti-inflammatory cytokine, it also plays a proinflammatory role. For instance, TGF-β promotes the differentiation of Th17 cells, which in turn releases cytokines including IL-22 and IL-17 that are proinflammatory in nature. Hence, the TGF-β also indirectly acts as a proinflammatory cytokine, depending on the microenvironment [106].

### Role of interleulin-10 (IL-10)

IL-10 is a significant mediator for the interactions between microglia and astrocyte in the CNS. Astrocytes express IL-10R1, and upon binding of IL-10 to IL-10R1 produce TGF-β. Then, the astrocytic TGF-β reduces the microglia activation [107]. The endothelial cells, as one important components of the BBB, stimulate microglia to produce IL-10 and iNOS under the deprivation of oxygen and glucose. In contrast, astrocyte, also a part of the BBB, does not alter the level of inflammatory cytokines [108]. Although IL-10 is an anti-inflammatory cytokine, some reports show that IL-10 might enhance pathological alteration in AD [109, 110]. As reported by Chakrabarty *et al*., IL-10 inhibits the microglia phagocytosis ability, which results in Aβ accumulation and exacerbation of memory loss [109]. Blocking the microglia IL-10/Stat3 signaling pathway increases the extent of Aβ phagocytosis, which suggests that inhibiting IL-10 might be a potential direction in developing AD therapies [110]. In contrast, an increase in IL-10 expression, inhibits the glia immune response and improves neurogenesis to alleviate memory disturbance without Aβ alteration [111] (Fig. 3).

### Role of complement

The classically activated microglia produce cytokines (*e.g*., TNF-α, IL-1α, and C1q) and induce the A1 astrocyte form, which is a traditionally reactive phenotype. A decrease in A1 astrocytes activity causes a reduction in synaptogenesis and induction of neuronal and oligodendrocytes cell death [112-114]. A1 astrocytes are abundant in various human neurodegenerative diseases, including AD, HD, PD, and ALS [112]. Classically activated microglia produce early complement components (C1q, C1r, C1s, and C3); among them, C1q causes C3 cleavage [115]. LPS induces astrocyte polarization into the A1 phenotype, which typically expresses C3[112, 116]. The A1 astrocytes exhibit high C3 expression levels, and high C3 levels are present in some neurodegenerative diseases (*e.g.*, PD and AD). The C3 positive astrocytes are near the Cluster of Differentiation 68 (CD68) positive microglia/macrophages. Because CD68 is a typical marker of activated microglia/macrophages [112, 117], it is suggested that activated microglia produce proinflammatory cytokines and complement components to promote the formation of the A1 type of astrocytes. Further, the interactions between C3 from A1 astrocytes and C3a receptor (C3aR) in microglia result in the loss of microglia phagocytosis activity [73, 112, 118]. Clarke *et al*. report that A1 astrocytic gene expression also alters with age. Complement C3, complement C4B, and cytokine CXCL10 increase with age, especially in the hippocampus and striatum [118]. A1 astrocytes lose their typical astrocytic protective effects and, at the same time, promote the immune response. Combining these factors leads to neurotoxicity [112-114, 118]. Aβ induces microglia to produce IL-1α, TNF-α, and C1q. The Aβ induced microglia conditioned medium induces astrocyte polarization towards the A1 phenotype. Therefore, inhibiting microglial inflammatory activity is one of the routes to reduce the damaging effects due to A1 type astrocytes. For instance, NLY01, a glucagon-like peptide-1 receptor, reduces the production of IL-1α, TNF-α, and C1q in microglia, which results in the reduction of A1 astrocytes. NLY01 protects dopaminergic neurons and improves the behavior in a PD animal model through the microglia-astrocyte interactions to modulate astrocyte polarization [113]. Additionally, milk fat globule epidermal growth factor 8 inhibits the A1/A2 alteration through NF-κB inhibition and PI3K-Akt activation [114].

Interactions between microglia and astrocyte also play a significant role in physiological and pathological processes in the CNS. Astrocyte and microglia crosstalk, as demonstrated by Lian et al., depends on the complement pathway in an AD mouse model [73]. C3, a protein of the immune system, is closely connected to cellular signal mediators between astrocytes and other cells in the CNS. Astrocytes and microglia are the primary sources of C3 and C3aR [73]. High levels of C3 expression are present in the brain and cerebrospinal fluid (CSF) of AD patients [119], and high levels of C3 affect the microglial inflammatory and phagocytotic functions [73]. IFN-γ treatment increases microglial C3 expression [115]. In Aβ-treated astrocytes and aged AD APP/PS1 mouse model, the astrocytic C3 level elevates, suggesting that the astrocytic complement system is involved in amyloid pathology in AD [73, 120]. Indeed, associated with Aβ formation, the activated astrocytes express high levels of C3 [120, 121], and exposure to Aβ triggers astroglial C3 release [119, 120]. As stimuli, Aβ increases the microglial immune response and reduces the ability of the microglia to clear the Aβ in AD [122].

C3 and its receptor C3aR are present within and around Aβ cerebral plaques and contribute to the phagocytosis and clearance of fibrillar Aβ by microglia in AD. C3 activates the microglia C3aR to mediate β-amyloid pathology and neuroinflammation [73, 115]. Upon triggering by C3, neurons also express C3aR [73, 120]. Interactions between neuronal C3aR and astrocytic C3 cause an inflammatory response leading to synaptic density loss and dendritic morphological alternation [120]. The C3-C3aR pathway activation reduces Aβ clearance, while the treatment with a C3aR antagonist SB290157 increases the Aβ clearance in C3 treated microglia [73]. In AD mice, the astrocytic C3-C3aR pathway results in Aβ accumulation via the NF-κB pathway [73], while astrocytes elevate C3 via the NF-κB pathway in AD mice. The NF-κB pathway produces various functional downstream factors, such as inflammatory factors and oxidative molecules [123, 124]. IκBα elimination causes C3 up-regulation [73, 120].

### Role of the microbiota-gut-brain axis in AD

#### Disorders of gut bacterial in AD

Alteration of the bacterial composition is one of the key features of the gut-brain axis (GBA) dysbiosis. The subsequent gut barrier disruption and systemic inflammation trigger neuroinflammation, BBB impairment, neural damage, and neurodegeneration [83]. Analyses of samples of early- and late-onset AD, using next-generation sequencing, show that self-antigen load may cause pathogenesis of AD because of the neuroinflammation [125]. For example, activation of HMGB1 signaling pathway is a causative factor of neuroinflammation in AD progression [126]. Alcohol-mediated neuroinflammation is also associated with AD and gut dysbiosis [127]. Substantial accumulating evidence supports the relationship between the MGBA and AD pathology, which includes the typical pathological alteration in the formation of extracellular Aβ plaques and intracellular neurofibrillary tangles. Further, in AD mice, the presence of the beneficial bacterial family *Bifidobacteriaceae* decreases and the pathogenic bacterial family *Proteobacteria* increases, at the phylum level, within the microbiota [83]. While in AD patients, negative correlations exist between bacterial diversities and concentrations of CSF Aβ biomarkers [128].

**Table 2:** Different Spectrum of Microbiota and Microglia-Astrocyte Cross Talk in Alzheimer’s Disease.

Microbiota	Pathology/Disorder	Mediators	Function/Animal Model	Ref.
Decreased *Firmicutes, Verrucomicrobia, Proteobacteria and Actinobacteria* Increased *Bacteriodetes and Tenericutes*	AD-like pathology	Indirect microglia-dependent actions	APPSPS1 (8 months old) mice	[129]
*Lactobacillus johnsonii Muribaculum*	Aggravation of neuroinflammatory response	Activated microglia and reduced motor activity	3xTg-AD (FMT-AD) mice	[130]
*Lactobacillus reuteri*	Neuroinflammation	Ahr signaling in astrocytes and microglia	GFAP-Cre, and Ahr^fl/fl^ mice	[86]
*Toxoplasma gondii*	Amelioration of β-amyloidosis	Ly6C^hi^ Monocytes	AD mice	[131]
*Sutterella (Betaproteobacteria)*	AD pathology	Inflammation	Tg-APP/PS1 Transgenic mice (6 months old)	[132]
*Erysipelotrichaceae*			Tg-APP/PS1 Transgenic Mice (24 months old)	
*Bacteroides fragilis*	Plaque deposition	*Bacteroides* colonization	Tg 2576 transgenic mice	[133]
*Helicobacter pylori*	Aβ accumulation	Outer membrane vesicles	*AppNL-G-F* AD mice	[134]
*Porphyromonas gingivalis and Paenalcaligenes hominis*	AD-like pathology	Outer membrane vesicles	C57BL/6J mice (14 months old)	[135]
*Paenalcaligenes hominis*	Cognitive function-impaired disorders, such as AD	Extracellular vesicles through blood and vagus nerve	Germ -free C57BL/6J mice	[136]
*Porphyromonas gingivalis, Fusobacterium nucleatum, Borrelia burgdorferi and Chlamydia pneumoniae*	AD pathology	Multiple pathogens Aβ fibrils and Tau tangles	AD mice	[137]
*Aerococcus, Jeotgalicoccus, Blautia, Pseudomonas, Clostridiale and Ruminococcaceae*	Accumulation of Aβ plaque	Extracellular vesicles	Tg-APP/PS1 transgenic Mice	[138]
*Lachnospiraceae and S24-7*	Reduction in Aβ deposition	Microglia and Astrocytes	Aged APPSWE/PS1ΔE9 mice	[139]
*Akkermanisa ans S24-7*	AD pathology	Engraftment	APP/PS1 Transgenic Mice	[140]
*Bifidobacterium bifidum Lactobacillus plantarum*	Reduced Aβ accumulation and enhanced cognitive function	FMT	AD Rat model	[141]
*Proteobacteria, Verrucomicrobia, Akkenrmansia, Desulfovibrio, Bacteroidetes and Alloprevotella*	Alzheimer’s disease like-pathogenesis	FMT	APP/PS1 Transgenic Mice	[142]

In humans, eating habits affect the composition of gut microbiota and the microbiome plays an important role in aging and age-dependent disease. A double-blind clinical study, with mild cognitive impairment (MCI, the stage between the expected decline in memory and thinking that happens with age and the more serious decline of dementia) patients, found that the composition of the gut microbiota changes with 2 types of diets: 1) the American Heart Association Diet (AHAD), which consists of a low-fat and higher-carbohydrate meal, and 2) the Modified Mediterranean-Ketogenic Diet (MMKD), which has slightly higher carbohydrate consumption compared to the original ketogenic diet to permit increased intake of vegetables and fruits while emphasizing fats and proteins derived from healthy sources such as olive oil and fish [143]. The AHAD increases *Mollicutes* while the MMKD increases the abundance of *Enterobacteriaceae* and *Akkermansia* but decreases *Bifidobacterium* and *Lachnobacterium* [143]. MMKD, associated with increased CSF Aβ 42 and decreased tau, appears to suppress cognitive decline and improve CSF biomarkers in adults who were at risk for AD [143].

#### Proteobacteria and LPS

*Proteobacteria* is the main proinflammatory bacteria, causing various inflammatory diseases, *e.g*., diarrhea [144]. *Escherichia coli* is one of the members of the Gamma *Proteobacteria Enterobacteriaceae* family, and the inflammatory agent LPS is one of the constituents of its outer wall [145]. In AD patients, the blood LPS levels are 3-fold higher than that of healthy individuals. In addition, in the brain specimens of AD patients, results from 16s rRNA sequencing reveal that over 70% of bacteria are LPS+ α-proteobacteria [146]. In Wistar rats, LPS impairs spatial memory and causes hippocampal neuronal apoptosis [147]. CD14, an LPS receptor, enhances the phagocytosis of Aβ in microglia. Furthermore, in AD patients but not in control human subjects, a pronounced CD14 immunoreactivity on parenchymal microglia spatially correlates to characteristic AD lesion sites [148]. In AD pathology, LPS plays a bilateral role in having inflammatory effects while at the same time initiating the pathological process via clearing amyloid deposits. Injection of LPS into the brains of APP transgenic mice leads to the clearance of some pre-existing amyloid deposits. In a transgenic AD model, which contains the P301L mutation along with elevated levels of tau accumulation and production of Ser199/202.

TLRs are expressed on cell surface and in the intracellular space (*e.g.*, endosomes) by many immune cells, including monocytes and macrophages, inside and outside the CNS. Within the CNS, in a similar way like macrophages, microglia also express TLRs, however, the expression of TLRs in astrocytes and their participation in the CNS inflammatory responses is still in debate. It is generally considered that astrocytes do not express TLRs. However, the expression of TLRs in astrocytes occurs following the depletion of microglia [149]. Apparently, in some cases, astrocytes do express TLRs but their expression after binding to ligands (*e.g.*, LPS) does not trigger downstream signaling cascade, which usually involves key genes such as MyD88, NFB, TRIF, IRF3. In contrast, microglia express TLRs in response to LPS, DAMPs, and PAMPs as well as by IL-1α, TNFα, IL-6 and other proinflammatory cytokines, which indirectly induce astrocytes to transform to reactive or neurotoxic astrocytes. Most importantly, the crosstalk between the microglia and astrocytes is essential for inflammatory responses, which transpires through TLRs-mediated mechanisms.

LPS injection into the frontal cortex and hippocampus increases the expression levels of phosphorylation at Ser199/202 and Ser396 of tau protein, supporting the roles of proinflammatory LPS stimuli in promoting tau phosphorylation [150]. In contrast to the association between some proinflammatory bacteria and AD pathology, other gut bacteria might be protective. For example, probiotics are beneficial in protecting neurons against neurodegeneration. In AD mice, consumption of *Bifidobacterium*, a probiotic microorganism, improves the Y maze test outcomes, suggesting their potential roles in improving behavior and physiological processes of AD. The decrease in hippocampal inflammation and Aβ accumulation associate with the inhibition of cognitive AD impairment [151].

#### Short-chain fatty acids (SCFAs)

Small molecule SCFs in ones' diet modulate the composition of the gut flora in AD patients. A negative correlation exists between the concentrations of fecal SCFs (propionate and butyrate) and the CSF Aβ-42 concentration in MCI patients [143]. In individuals partaking in the MMKD diet, propionate and butyrate levels elevate, while lactate and acetate reduce. However, in patients with the AHAD diet, acetate and propionate levels increase, along with butyrate concentration decreasing [143]. SCFAs inhibit the Aβ1-40 interactions, which is key to the formation of Aβ1-40 dimers and trimers [152]. In fact, Aβ1-40 or Aβ1-42 peptide interactions are directly observed in vitro [153]. SCFAs also regulate inflammation, *e.g.*, by regulating leukocytes and endothelial cells through the G protein coupled-receptor and histone deacetylase signaling pathways, SCFAs regulate the secretion of cytokines such as IL-10, IL-2, and TNF-α, as well as the leukocyte migrations [154]. In AD patients, leukocyte activation and the increase of proinflammatory factors are two key AD inflammatory events. Additionally, infrared spectroscopic analysis of peripheral mononuclear leukocytes in mild and moderate AD patients are distinguished from healthy controls [155].

### Probiotics

Probiotics act on various benefits for many host organs, which is one of the important compositions of the gut-brain axis. Probiotics relieve some pathological processes in AD. Prebiotics as well as probiotics are used as preventive medicines to improve cognitive decline in AD, which can be caused by the alteration of commensal microbiota in the gut and dysfunction in gut-brain axis. Consumption of prebiotics and probiotics (synbiotics) together promote beneficial bacterial community in the gut. Recent clinical trials demonstrate the positive effects of probiotics for AD with a slowing down of disease progression [141]. For example, in a randomized controlled clinical trial, co-supplementation of a probiotic with selenium, in 79 patients, improves cognitive as well as metabolic functions. In other studies, preclinical and clinical, the results demonstrate that *Bacillus* or *Bifidobacterium breve* strain A1 metabolizes acetate, butyric, isovaleric, propionic, and valeric acids and promotes cognitive functions and inhibits deposition of Aβ *via* microglia and astrocytes mediated decreased inflammation in AD mice and AD patients [156]. Probiotics reduce oxidative stress and proinflammatory processes in AD rats [157]. The probiotics also restore the disorder of metabolism in AD mice. Oral probiotics enhance glucose uptake by affecting some associated metabolic molecules, such as glucose transporters, in 3xTg-AD [158]. The probiotics not only reduce inflammation and debris accumulation, but also decrease gut inflammation and gut permeability [159].

### Xenobiotic receptors

Various studies report the multifaceted roles of the xenobiotic receptors, which include the AHR, pregnane X receptor (PXR), and constitutive androstane nuclear receptor (CAR), in the MGBA. Firstly, AHR acts as a mediator in the bi-directional communication between the brain and gut microbiota [160]. In the AD mice model, AHR activates occludin and claudin 1 and 4, thereby enhancing and restoring BBB integrity [161]. Secondly, in activated astrocytes, AHR suppresses neuroinflammation via dietary supplementation with the microbial metabolites of Trp (AHR ligands) [86]. PXR is a nuclear transcription factor referred to as the master maintainer of the barrier integrity in both the intestine and brain. While PXR-deficient mice exhibit “leaky” intestine symptoms. Ligand-activated PXR prevents leaky gut and maintains the intestinal epithelial barrier by stimulating the synthesis of junctional proteins [162]. Moreover, PXR knockout mice show decreased expression of tight junction protein ZO-1 in the cortex and cortical microvessels [163]. Activation of PXR *in vivo* tightens the BBB, which is mediated by microbiota derived-ligands. Thirdly, increasing evidence provides a basis to support the potential of CAR-mediated signaling between the gut and brain. Activation of CAR promotes mucosal healing by altering the natural milieu of the microbiota. Notably, CAR affects structural factors in the brain and the behavior of mice as CAR-null mice show impairment in recognition memory accompanied by neuronal and structural defects in the hippocampus and cortical area. Moreover, tight junction protein ZO-1 expression reduces in the cortical and hippocampal microvessels of the mice [164].

### Trp controls the microglia-astrocyte crosstalk in AD - involvement of the microbiota-gut-brain axis

People with C3 deficiency are susceptible to bacterial infection [165]. Like other immune substances, Trp metabolism affects the complement system, in which dietary Trp levels modulate the expression of C3 [73].

For Trp metabolites, signaling through AHR constitutes the interface of MGBA. Ligands derived from microbial Trp metabolism play critical roles in intestinal homeostasis [166]. The gut flora strongly influences Trp metabolism. For instance, tryptophanase from *Escherichia coli* generates indole from Trp [167]. Several bacteria species convert Trp into indole and indole derivatives, including IPA, tryptamine, 3-methylindole (also known as skatol), indole-3-aldehyde (I3A), indoleacetic acid, indoleacrylic acid, indoleethanol, indolelactic acid [168]. Some of these derivatives are AHR ligands. For instance, *Lactobacillus* species metabolize Trp into I3A, which activates AHR in intestinal immune cells and induces cytochromes P450 1A1 and P450 1B1 expression [169]. Indole itself acts as a ligand for AHR and serotonin is an AHR ligand. Indole decreases mucosal inflammation and injury in animals [170].

As a dioxin-binding protein, AHR is initially recognized as a regulator of dioxin and AHR causes immunosuppression after binding with dioxin. As a nuclear protein, AHR is a highly conserved transcription factor that affects aging and age-associated diseases (*e.g.*, AD) by regulating many target genes [171]. KYN is one of the endogenous ligands of the AHR. Some natural ligands like KYN and KYNA (an AHR agonist with higher titer) also trigger AHR to affect differentiation and immunity [172]. For example, Benzo(a)pyrene, one of the AHR ligands, causes the alteration of the levels of neurotransmitters and leads to apoptosis as part of neurodegeneration [173]. AHR is involved in innate and adaptive immune responses [174].

**Figure 4.**
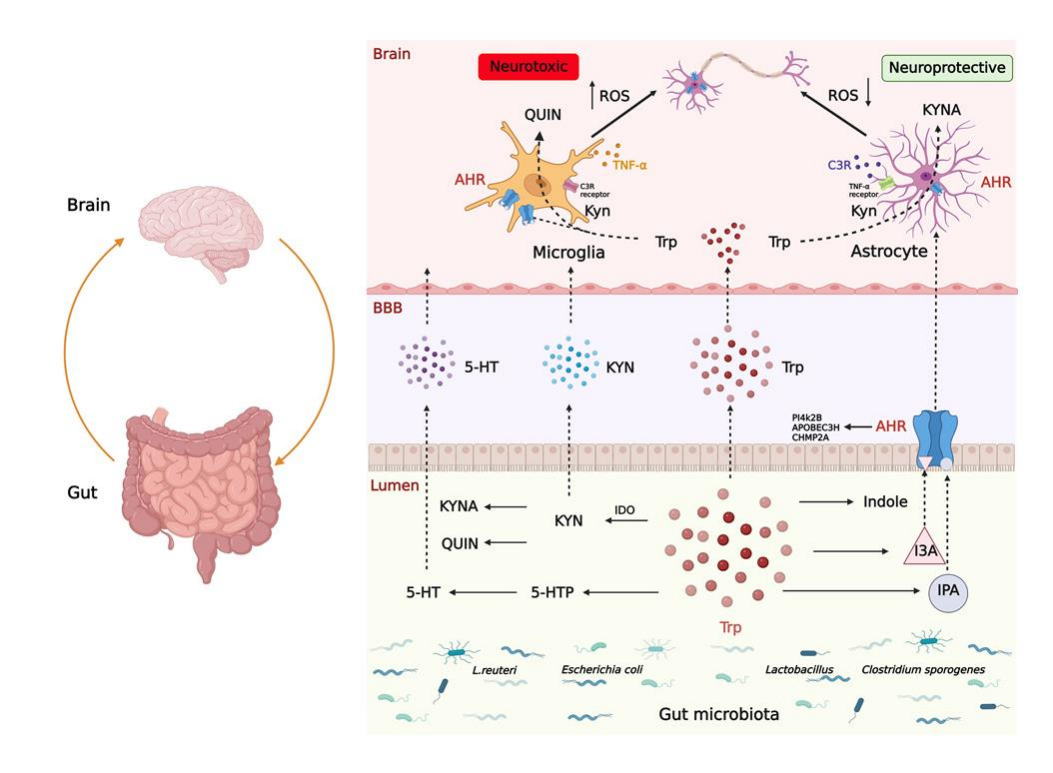
Tryptophan participates in microglia and astrocyte crosstalk in AD with the involvement of MGBA. The tryptophan (Trp) metabolism regulates the interaction between microglia and astrocyte. Molecules, such as C3, TNF-α, control the interaction. The C3 originates from microglia-primed astrocytes and the TNF-α is released from astrocytes-primed microglia. The interaction of microglia and astrocyte affects their functions (*e.g.*, phagocytosis and generation of ROS) that are essential pathological processes in AD. The metabolism of gut flora produces small molecules, such as Trp. The metabolites of Trp and Trp affect the interaction between microglia and astrocyte via ROS pathway. Trp regulates three major metabolic pathways (*e.g.*, 5-HT, KYN, and AHR ligand pathways). Alteration in gut microbiota composition, Trp synthesis and its related metabolites lead to change in the pattern of microbiome within the gut. In the brain, Trp acts on the AHR to stimulate the production of QUIN and KYNA. The activation of microglia and astrocyte through AHR increases and decreases ROS through the release of QUIN and KYNA, resulting in neurotoxic and neuroprotective effects, respectively. The top three CMap gene connections of AHR are PI4KIIB, APOBEC3, and CHMP2A. Trp controls the microglia-astrocyte crosstalk in AD with the involvement of MGBA mechanisms. This figure was created with BioRender.com.

AHR’s involvement in the immune response is also seen in its Connectivity Map, or CMap, using L1000 [175]. CMap is a resource that assists in identifying relationships between diseases, genes, and therapeutics. The top three CMap gene connections of AHR are shown in Table 3, and are PI4KIIB, APOBEC3, and CHMP2A. Any connection score over 95 means the connection is strong. PI4KIIB is implicated in the activation of early T cells via the CD3-T Cell Receptor signaling pathway [176]. The APOBEC3 gene is an interferon-stimulated gene whose expression increases in response to various stimuli, and thus the activation of innate immune responses that occurs upon infection, with many different viruses, could lead to increased APOBEC3 activity [177] (Fig. 4). Silencing of ESCRT-III-protein CHMP2A results in spontaneous necroptosis. As necroptosis actively shapes the immune response, ESCRT-III indirectly controls the immunogenicity of necroptotic dying cells [178].

**Table 3:** Genes connected with AHR found using L1000.

Gene Name	Connection Score	Description
PI4k2B	98.45	Phosphatidylinositol kinases
APOBEC3H	96.85	Apolipoprotein B mRNA editing enzymes
CHMP2A	95.78	Charged multivesicular body proteins

AHR also plays a role in the glia immune response in both proinflammatory and anti-inflammatory processes. A Trp deficient diet induces gut microbiota dysbiosis and increases systemic inflammation [179]. Trp deficiency or IDO elevation activates the NF-κB pathway, which results in oxidative damage and proinflammatory responses [180, 181]. The basic function of microglia in AD is to clear pathological debris and control the inflammatory response. The increase of proinflammatory signaling inhibits the microglia’s ability to clear Aβ, while AHR might elevate the microglia’s phagocytosis capacity via inhibition of the microglia's proinflammatory responses [182, 183]. Microglial AHR activation, as reported by Lee *et al*., exerts bi-directional effects: the regulation of LPS-induced microglia inflammation depends on the availability of external AHR ligands [184]. In the absence of AHR ligands, LPS activates AHR via the MER1/2 pathway, and AHR knockout inhibits the microglia response, leading to proinflammation and neurotoxicity. As a result, in the absence of AHR ligands, the activation of microglial AHR exerts proinflammatory effects. On the contrary, in the presence of AHR ligands, the activation of AHR attenuates the LPS-induced microglia’s proinflammatory function by reducing LPS-induced AHR binding to the iNOS and TNF-α [184].

LPS and IFN-γ increase the AHR recruitment, while AHR also inhibits the NF-κB to reduce proinflammatory factors (eCCL2, Csf2, and NOS2) [86]. Astrocytic AHR limits CNS inflammation, as demonstrated in AHR^fl/fl^ mice. The AHR deficient astrocytes produce more proinflammatory substances, such as CCL2 and IL-6, which promote the inflammatory response and immune cell recruitment [86]. These chemokines and cytokines are essential signaling factors in AD. Astrocytes also express these molecules and their receptors, which trigger the inflammatory chain reaction [100, 182].

Trp also affects the microglia-astrocyte crosstalk. Trp metabolites, such as IPA and I3A, activate microglia, which then initiate signaling through the AHR in astrocytes (Fig. 4). The dietary Trp also induces microglia to produce TGF-α and VEGF-β, and finally modulates the astrocyte transcriptional program through the AHR-mediated signaling [88]. The gut microbacterium *L. reuteri* degrades Trp to indole, which is then converted into many other derivatives, including IPA, I3A, and indole-3-sulfate. These indole derivatives cross through the BBB and interact with AHR in the CNS [86, 185].

Inhibiting either Trp synthesis or its uptake causes a decrease in Trp concentrations in the brain, which in turn results in emotional and behavioral changes [34]. Both neurons and glia express AHR, and the AHR ligands regulate AHR levels [86, 186]. AHR participates in various physiological and pathological processes that are highly relevant to intestinal homeostasis and CNS diseases, including AD. AHR serves as a critical node in MGBA through Trp metabolism in microbiota and related brain signaling by Trp metabolites [166].

Activation of AHR in neurons alters the caspase-3 activation, ROS expression, and LDH release [186]. As part of the growing interest in MGBA, AHR activation is also a potential therapeutic target to regulate the CNS inflammation [166]. Trp metabolites activate microglia, which then modulate astrocytes through AHR signaling to regulate the CNS inflammation [187]. The deficiency of astrocytic AHR leads to the expression of proinflammatory substances (*e.g*., IL-6, CXCL10, and CCL2 [86]. Microglial AHR signaling also regulates the immune response in the CNS [184]. Deletion of AHR increases microglial activation and affords increased levels of neuroinflammation and neurodegeneration [188, 189]. In microglial AHR deletion mice, astrocytes increase their expression of proinflammation and neurodegeneration genes (*e.g.*, IL-1β, CCL2, and NOS2) [88] highlighting microglia and astrocyte crosstalk.

In summary, some microbiota species (*e.g.*, *Lactobacillus ssp*., *Bifidobacterium ssp*., and *Peptrostreptococcus Russellii*) produce some AHR ligands (*e.g.*, Trp derivative IPA and indole) to activate AHR [190]. The microglial AHR activation exerts proinflammatory effects and causes increased neuroinflammation and neurodegeneration. In contrast, astrocytic AHR activation limits CNS inflammation. In AD animal models, the activation of AHR in neurons alters the levels of apoptosis and oxidative stress, which alleviate cognitive deficits. Thus, AHR activation may offer solutions to developing AD therapies.

MGBA is a bidirectional link between the CNS and the enteric nervous system (ENS). ENS is a complex neuronal network. The gut saprophytic microflora can communicate with both the ENS and the CNS, *e.g.*, the neurotransmitter signaling by Trp metabolites through MGBA. Serotonin is a neurotransmitter in both the CNS and the ENS. Both the brain and the gut produce serotonin. Some bacteria found in the gut microbiota synthesize serotonin [191]. Mucosal serotonin incites intestinal inflammation, and the reduction of serotonin and KYNA levels in the intestine is related to intestinal homeostasis disturbances [192].

### Summary

Trp plays a vital role in regulating the GBA and is involved in neurodegenerative diseases such as AD, PD [193, 194], HD [195] as well as dementia [196]. Glia, especially the interaction between different glia, influence the pathological process of neurodegeneration. In this review, focusing on AD, we discuss the Trp metabolism and its roles in the glia pathology of AD, where Trp and its metabolites enter the CNS and modulate astrocytes-microglia crosstalk (Fig. 4). The importance of communication between glia and neuron is recognized. However, the connections between Trp and neurodegeneration remain elusive. Potential future research directions include a more comprehensive understanding of the roles of Trp and Trp metabolites in AD and aging biology; the AHR activation; targeting Trp metabolism and AHR in developing new therapies for AD; and the development of probiotics by modulating Trp metabolism. Continued preclinical and clinical studies will shed light on the crosstalk between microglia and astrocyte and MGBA. Such studies will elucidate the important role(s) of Trp as well as rationales for new therapeutic targets and strategies to treat AD, which afflicts millions of individuals worldwide.
